# Update to the study protocol for a randomized controlled trial comparing mindfulness-based cognitive therapy with maintenance anti-depressant treatment depressive relapse/recurrence: the PREVENT trial

**DOI:** 10.1186/1745-6215-15-217

**Published:** 2014-06-10

**Authors:** Willem Kuyken, Sarah Byford, Richard Byng, Tim Dalgleish, Glyn Lewis, Rod Taylor, Edward R Watkins, Rachel Hayes, Paul Lanham, David Kessler, Nicola Morant, Alison Evans

**Affiliations:** 1 Mood Disorders Centre, School of Psychology, Perry Road, University of Exeter, Exeter EX4 4QG, UK; 2 King’s College London, Centre for the Economics of Mental and Physical Health, Box PO24, Institute of Psychiatry, De Crespigny Park, London SE5 8AF, UK; 3 Primary Care Group, Peninsula College of Medicine and Dentistry, University of Plymouth, N32, Tamar Science Park, Drake Circus, Plymouth PL4 8AA, Devon; 4 Medical Research Council Cognition and Brain Sciences Unit, Chaucer Road, Cambridge CB2 7EF, UK; 5 School of Social and Community Medicine, Oakfield House, Oakfield Grove, University of Bristol, Bristol BS8 2BN, UK; 6 10 Alexander Close, Clifton SG17 5RB, Bedfordshire; 7 Department of Psychology, University of Cambridge, Downing Street, Cambridge CB2 3 EB, UK

**Keywords:** Mindfulness-based cognitive therapy, Randomised controlled trial, Depression, Antidepressant, Trial protocol

## Abstract

**Background:**

Depression is a common and distressing mental health problem that is responsible for significant individual disability and cost to society. Medication and psychological therapies are effective for treating depression and maintenance anti-depressants (m-ADM) can prevent relapse. However, individuals with depression often express a wish for psychological help that can help them recover from depression in the long-term. A recently developed treatment, mindfulness-based cognitive therapy (MBCT), shows potential as a brief group program for people with recurring depression.

This trial asks the policy research question; is MBCT with support to taper/discontinue antidepressant medication (MBCT-TS) superior to m-ADM in terms of: a primary outcome of preventing depressive relapse/recurrence over 24 months; and secondary outcomes of (a) depression free days, (b) residual depressive symptoms, (c) antidepressant medication (ADM) usage, (d) psychiatric and medical co-morbidity, (e) quality of life, and (f) cost effectiveness? An explanatory research question also asks whether an increase in mindfulness skills is the key mechanism of change.

The design is a single-blind, parallel randomized controlled trial examining MBCT-TS versus m-ADM with an embedded process study. To answer the main policy research question the proposed trial compares MBCT-TS with m-ADM for patients with recurrent depression. Four hundred and twenty patients with recurrent major depressive disorder in full or partial remission will be recruited through primary care.

**Results:**

Depressive relapse/recurrence over two years is the primary outcome variable. Analyses will be conducted following CONSORT standards and overseen by the trial’s Data Monitoring and Safety Committee. Initial analyses will be conducted on an intention-to-treat basis, with subsequent analyses being per protocol. The explanatory question will be addressed in two mutually informative ways: quantitative measurement of potential mediating variables pre- and post-treatment and a qualitative study of service users’ views and experiences.

**Conclusions:**

If the results of our exploratory trial are extended to this definitive trial, MBCT-TS will be established as an alternative approach to maintenance antidepressants for people with a history of recurrent depression. The process studies will provide evidence about the effective components which can be used to improve MBCT and inform theory as well as other therapeutic approaches.

**Trial registration:**

Trial registered 7 May 2009; ISRCTN26666654.

## Update

This update relates to the study protocol for a randomized controlled trial comparing mindfulness-based cognitive therapy with maintenance anti-depressant treatment in the prevention of depressive relapse/recurrence: the PREVENT trial. This update should be read in conjunction with the original protocol publication [1].

### Reworded research question

To ensure that readers clearly understand that this trial is not a direct comparison between antidepressant medication (ADM) and Mindfulness-based cognitive therapy (MBCT), but ADM versus MBCT plus tapering support (MBCT-TS), the primary research question has been changed following the recommendation made by the Trial Steering Committee at their meeting on 24 June 2013. The revised primary research question now reads as follows: ‘Is MBCT with support to taper/discontinue antidepressant medication (MBCT-TS) superior to maintenance antidepressant medication (m-ADM) in preventing depression over 24 months?’ In addition, the acronym MBCT-TS will be used to emphasise this aspect of the intervention.

### Subgroup analysis high versus low childhood adversity

The Trial Management Group met on 8 May 2013 and discussed the early dissemination via personal communication of Williams *et al.* (subsequently published [2]) comparison of MBCT, cognitive psychological education and treatment-as-usual. This study suggested that MBCT compared with the other two conditions confers a significant protection against relapse for participants with increased vulnerability due to a history of childhood trauma. It was agreed at this meeting that an additional subgroup analysis should be added to our statistical analysis plan to examine the potential difference in treatment outcome as related to childhood adversity. This will be undertaken by examining the interaction of childhood abuse with treatment allocation on the primary outcome at 24 months. The statistical analysis plan was updated accordingly on 15 May 2013.

### Ethical approval and trial governance

We have received multicenter ethical approval (South West Exeter Research Ethics Committee, 09/H0206/43) and local research governance approval for all sites (NHS Devon covering Exeter and Mid-and North-Devon, PCT0739; NHS Bristol, covering Bristol site, 2010-004, NHS Plymouth and NHS Torbay, PLY-TOR001 covering South Devon site). The study personnel, management group, and independent Trial Steering Committee will ensure that the study is conducted within appropriate NHS and professional ethical guidelines, ensuring that Good Clinical Practice guidelines are observed at all times. This trial has been approved by the Medicines and Healthcare products Regulatory Agency (EudraCT number 2009-012428-10). All participants gave full informed consent.

## Abbreviations

ADM: Antidepressant medication; m-ADM: Maintenance antidepressants; MBCT: Mindfulness-based cognitive therapy; MBCT-TS: MBCT with support to taper/discontinue antidepressant medication.

## Competing interests

The authors declare that they have no competing interests.

## Authors’ contributions

WK, SB, RB, TD, GL, RT, ERW, PL, DK and NM conceived and designed the study and obtained funding. AE is the lead MBCT therapist responsible for training and supervision. RH is the trial manager. WK drafted the manuscript and all other authors contributed to editing of the final manuscript. All authors contributed to, and approved, the final manuscript.
